# Management of chronic sensitization, from drugs to physical therapy

**DOI:** 10.1186/1129-2377-14-S1-O10

**Published:** 2013-02-21

**Authors:** Nathalie Roussel, Mira Meeus, Liesbeth Daenen, Jessica Van Oosterwijck, Patrick Cras, Jo Nijs

**Affiliations:** 1 Department of Rehabilitation Sciences and Musculoskeletal Physiotherapy, Artesis College, Antwerp, Belgium

## 

Over the past decades, scientific understanding of unexplained chronic pain disorders has increased substantially. Several chronic musculoskeletal pain disorders may be explained by alterations in central nervous system processing. More specifically, the responsiveness of central neurons to input from unimodal and polymodal receptors is augmented, resulting in a pathophysiological state corresponding to central sensitization, characterized by generalized or widespread hypersensitivity. Central sensitization encompasses altered sensory processing in the brain, impaired functioning of top-down anti-nociceptive mechanisms, and (over)activation of top-down and bottom-up pain facilitatory pathways which augment nociceptive transmission. Importantly, a different ‘pain signature’ arises in the brain of those with chronic musculoskeletal pain and central sensitization.

Given the increasing evidence supporting the clinical significance of central sensitization in those in a wide variety of disorders, including chronic whiplash associated disorders, chronic tension-type headache and migraine among others, the awareness is growing that desensitising the central nervous system should be a treatment target in these patients. Besides pharmacological options, rehabilitation (consisting of pain physiology education, stress management and exercise therapy) and neurotechnology options (e.g. Transcranial magnetic stimulation, TENS, virtual reality) offer interesting perspectives.

